# Short, frequent high-intensity physical activity breaks reduce appetite compared to a continuous moderate-intensity exercise bout

**DOI:** 10.1530/EC-22-0259

**Published:** 2022-12-22

**Authors:** Benjamin D Maylor, Julia K Zakrzewski-Fruer, Charlie J Orton, Daniel P Bailey

**Affiliations:** 1 Institute for Sport and Physical Activity Research, School of Sport Science and Physical Activity, University of Bedfordshire, Bedford, UK; 2 Leicester Diabetes Centre, University of Leicester, Leicester General Hospital, Leicester, UK; 3 Centre for Physical Activity in Health and Disease, Brunel University London, Uxbridge, UK; 4 Division of Sport, Health and Exercise Sciences, Department of Life Sciences, Brunel University London, Uxbridge, UK

**Keywords:** sedentary behaviour, activity breaks, exercise, satiety, appetite hormones

## Abstract

A single exercise session can affect appetite-regulating hormones and suppress appetite. The effects of short, regular physical activity breaks across the day on appetite are unclear. This study investigated the effects of breaking up sitting with high-intensity physical activity vs a single bout of moderate-intensity exercise and prolonged sitting on appetite control. In this randomised crossover trial, 14 sedentary, inactive adults (7 women) completed 3, 8-h experimental conditions: (i) prolonged sitting (SIT); (ii) 30 min of moderate-intensity exercise followed by prolonged sitting (EX-SIT), and (iii) sitting with 2 min 32 s of high-intensity physical activity every hour (SIT-ACT). Physical activity energy expenditure was matched between EX-SIT and SIT-ACT. Subjective appetite was measured every 30 min with acylated ghrelin and total peptide-YY (PYY) measured hourly in response to two standardised test meals. An *ad libitum* buffet meal was provided at the end of each condition. Based on linear mixed model analysis, total area under the curve for satisfaction was 16% higher (*P* = 0.021) and overall appetite was 11% lower during SIT-ACT vs EX-SIT (*P* = 0.018), with no differences between SIT-ACT and SIT. Time series analysis indicated that SIT-ACT reduced subjective appetite during the majority of the post-lunch period compared with SIT and EX-SIT, with some of these effects reversed earlier in the afternoon (*P* < 0.05). Total PYY and acylated ghrelin did not differ between conditions. Relative energy intake was 760 kJ lower during SIT-ACT vs SIT (*P* = 0.024). High-intensity physical activity breaks may be effective in acutely suppressing appetite; yet, appetite-regulating hormones may not explain such responses.

## Introduction

A significant proportion of the population engages in high volumes of sedentary behaviour due to technological advancements, shifts in societal behaviour and increases in the number of office-based jobs (1). High levels of sedentary time are associated with an increased risk of overweight and obesity (2) and adverse health outcomes including cardiovascular disease, type 2 diabetes, and all-cause mortality (3, 4). Overweight and obesity result from a sustained positive energy balance (5); hence, it is important to identify interventions that increase energy expenditure without a consequent increase in energy intake to ensure the maintenance of an energy deficit. Despite growing interest in the health-enhancing effects of accumulating physical activity in short regular bouts to break up sitting (6), the effects of this pattern of physical activity on appetite are not well understood. This is important as a reduction in energy expenditure due to a single day of prolonged sitting is not accompanied by a reduction in appetite and may thus contribute to excess energy intake and weight gain (7).

It has consistently been demonstrated that moderate and high-intensity aerobic exercise performed in a single bout of >30 min can lead to an energy deficit due to suppressed subjective appetite, changes in circulating appetite-regulating hormones and/or no compensation in energy intake to account for the increased energy expenditure (8, 9). In the limited evidence base on breaking up sitting and appetite, Holmstrup, Fairchild, Keslacy, Weinstock, and Kanaley (10) observed reduced hunger in response to hourly, 5-min moderate-intensity physical activity bouts in the afternoon compared to prolonged sitting and a 1-h moderate-intensity exercise bout followed by prolonged sitting. Yet, there was no concomitant change in total peptide-YY (PYY) concentrations, which is an appetite suppressant (11). In contrast, breaking up sitting with 2-min brisk walking bouts every 20 min over 5.5 h significantly increased appetite-suppressing hormones (glucagon-like peptide 1 and total PYY), although subjective appetite responses were not evaluated (12). Another study did not find any subjective or appetite hormone responses to sitting interrupted with 2 min of light or moderate-intensity walking every 20 min over 5 h compared to prolonged sitting (13). Nevertheless, the increased energy expenditure of the physical activity bouts was not compensated for in a subsequent *ad libitum* buffet meal, thus resulting in an energy deficit of 600–1400 kJ. There is evidence to suggest that high-intensity physical activity performed in a single continuous bout may have a more pronounced effect on subjective appetite and appetite-regulating hormones than lower intensities (14, 15). In practical terms, inactive and sedentary individuals, who are target populations for physical activity promotion, may find it difficult to complete high-intensity physical activity in a single bout. Thus, investigating the appetite-regulating effects of breaking up prolonged sitting with short bouts of high-intensity physical activity across the day, which may be more achievable for individuals to complete, could be valuable to inform weight management strategies.

The aim of this study was to investigate the acute effects of breaking up sitting with hourly high-intensity physical activity breaks compared to a single continuous energy-matched moderate-intensity exercise bout and prolonged sitting on subjective appetite (primary outcome), appetite hormones, and energy intake (secondary outcomes).

## Materials and methods

### Study overview

This was a three-condition randomised crossover trial approved by the University of Bedfordshire Institute for Sport and Physical Activity Research Ethics Committee (approval number: 2015ISPAR004). Written informed consent was provided by each participant prior to any study procedures. Data collection took place at the University of Bedfordshire Sport and Exercise Science Laboratories. After preliminary measures, participants took part in three experimental conditions that were each 8 h in duration: (i) prolonged sitting, (ii) continuous moderate-intensity exercise followed by prolonged sitting, and (iii) sitting interrupted with high-intensity physical activity breaks.

### Participants

Participants were males and females aged 18–55 years and were of a mixed weight status. To be eligible, participants needed to self-report sitting for at least 7 h/day (based on evidence showing significantly increased risk of mortality above this threshold (16)) and engage in less than 150 min/week of moderate-to-vigorous physical activity; this was measured using the International Physical Activity Questionnaire (17). Participants were ineligible to take part if they were pregnant, had diabetes, were using glucose or lipid medication, had any know contraindications to physical activity, had a blood-borne disease, major illness or injury, or any allergies to the food and drink being provided in the study.

### Sample size

Sample size estimations were based on previous data (18). Based on a 10% within-group error variance, a within-person correlation of 0.6, 80% power, and α = 0.05, it was estimated that 12 participants would be required to detect a 10% difference in the primary outcome (subjective appetite). This value was inflated to 14 participants to accommodate for potential drop-out. Subjective appetite was the variable expected to have the smallest worthwhile change with a large amount of variability.

### Preliminary measures

Participants attended the laboratories to complete preliminary measures. Stature (Holtain Ltd., Crymych, Wales), body mass, and body composition (Tanita BC-418; Tanita Corp., Tokyo, Japan) were measured. Following this, participants completed an incremental exercise test on a motorised treadmill (Woodway PPS55Med-I, GmbH, Germany) to determine maximal oxygen uptake (V˙O_2max_). An online gas analysis system was used to measure expired air during the test (Cortex Metalyzer 3B, GmbH, Germany). Heart rate (HR) was measured using a Polar FS2 HR monitor (Polar Electro, Warwick, UK). Participants self-selected a starting speed that they felt they would be able to comfortably maintain for 30 min. This speed was subsequently increased by 1 km/h every 3 min until volitional exhaustion. V˙O_2max_ was taken as the highest V˙O_2_ value averaged over a 10 s period. At least two of the following end-point criteria were required for V˙O_2max_ to be considered as having been achieved: (i) plateau of V˙O_2_ despite increasing workload, (ii) Borg Rating of Perceived Exertion ≥18 (19), (iii) HR within 10 bpm of age-predicted maximum (220 – age), and (iv) respiratory exchange ratio >1.15 (20). A linear regression was produced using the average V˙O_2_ during the last 30 s of each stage, and the treadmill speeds that were estimated to elicit 60% and 85% V˙O_2_ reserve (V˙O_2_R) were determined for each individual participant. These speeds were used for the experimental conditions.

### Experimental protocol

**Figure 1:**
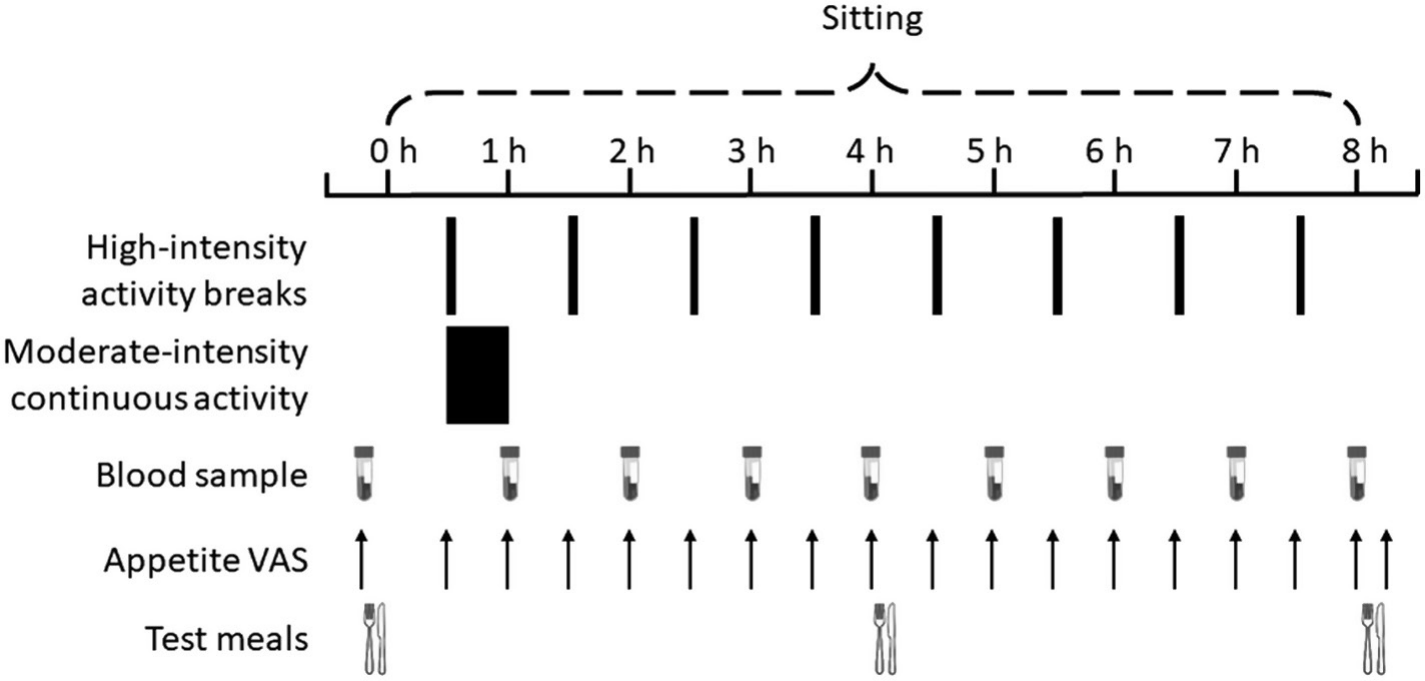
Schematic of experimental protocol. VAS, visual analogue scale.

The conditions were carried out in an incomplete counterbalanced order, which was pre-determined using the Latin square method. Due to the influence of the menstrual cycle on food intake (21) and appetite-regulating hormones (22), females completed experimental conditions during their self-reported follicular phase. This phase is typically not characterised by increases in appetite (21). The experimental conditions were thus separated as 6–35 days for women and 7–14 days for men. The 8-h experimental conditions, which are shown in Fig. 1., were:

1. Prolonged sitting (SIT): participants were seated throughout the condition.
2. Continuous moderate-intensity exercise followed by prolonged sitting (EX-SIT): participants completed 30 min of continuous moderate-intensity exercise at 60% V˙O_2_R, 30 min into the experimental condition, followed by prolonged sitting for the remainder of the condition.
3. Sitting interrupted with high-intensity physical activity breaks (SIT-ACT): participants completed 2 min 32 s bouts of high-intensity physical activity at 85% V˙O_2_R at 60-min intervals. The first bout took place 30 min into the condition. Eight bouts were thus completed with an accumulative duration of 20 min 16 s.

The decision to compare high-intensity physical activity breaks with a continuous moderate-intensity exercise session was based on previous appetite-related research fixing the intensity of the single exercise session compared with the short, frequent physical activity bouts (10). The protocol herein would thus provide novel evidence regarding the effects of high-intensity physical activity breaks. It was deemed inappropriate to compare high-intensity physical activity breaks to a single continuous high-intensity exercise session as sedentary inactive individuals are likely to find high-intensity physical activity difficult to complete in a single session, thus making the study ecologically valid. Physical activity energy expenditure was matched between EX-SIT and SIT-ACT on the assumption that 30 min of physical activity at 60% V˙O_2_R would have a similar energy consumption to 20 min 16 s of physical activity at 85% V˙O_2_R (23). The physical activity was thus completed in 2 min 32 s bouts in SIT-ACT so that it was equally spread across the 8 hourly bouts. The moderate and high-intensity physical activity bouts corresponded to a brisk walk to slow jogging pace.

Participants attended the laboratories in the morning following an overnight (>10 h) fast. Prior to the first experimental condition, participants completed a food diary detailing the times, type, and quantity of all food and liquid consumed the day before. Participants were asked to replicate this exact dietary intake the day before all subsequent visits, which was verbally confirmed upon arrival to the laboratory. They were also asked not to engage in any structured exercise or consume alcohol or caffeine for 48 h before the experimental conditions. An Actiheart monitor (CamNtech Ltd., Cambridge, UK) was worn during each condition to estimate physical activity energy expenditure. The device was calibrated individually for each participant using anthropometric and V˙O_2_ data (HR-energy expenditure relationship from the 3-min submaximal stages) collected during preliminary testing. The Actiheart device uses branched chain algorithms to calculate energy expenditure based on HR and tri-axial accelerometry and is validated for use in laboratory settings (24). Fasting measures of subjective appetite and appetite-regulating hormones were obtained before participants consumed a standardised breakfast. This meal comprised cornflakes and whole milk and was provided in quantities that corresponded to 15% of estimated individual daily energy requirements. This is in line with proposed definitions of a breakfast meal comprising ecologically valid food items (25, 26). The macronutrient composition was 55% carbohydrate, 30% fat, and 15% protein; the meal glycaemic index was 79. Energy requirements for each participant were estimated using the Mifflin equation (27) applying a physical activity factor of 1.4 to represent a sedentary day. Each experimental condition commenced once the last mouthful of the breakfast had been swallowed. A standardised lunch meal was consumed 4 h into the condition. This meal included the same ingredients and macronutrient composition as the breakfast meal to ensure a sufficiently high glycaemic challenge and to allow more direct comparisons of responses across the conditions but provided 30% of estimated daily energy requirements to reflect higher habitual energy intake at lunch compared with breakfast (28). In the first experimental condition, participants consumed water* ad libitum*. During subsequent conditions, the volume of water intake was replicated by providing equal amounts over the course of the day. Participants remained seated during the experimental conditions apart from when they completed the physical activity bouts or visited the lavatory. While sitting, they were free to work on a laptop computer, use their mobile phone or smart device, talk, or read books.

### Ratings of perceived appetite

During each condition, subjective feelings of hunger (’How hungry do you feel?’), satisfaction (’How satisfied do you feel?’), fullness (’How full do you feel?’), and prospective food consumption (PFC; ‘How much do you think you can eat?’) were reported using a validated paper and pen-based 100-mm visual analogue scale (VAS) (29). The VAS was completed in the fasted state and every 30 min during the experimental conditions. An overall appetite score was calculated as the mean value of the four appetite perceptions after inverting the values for satisfaction and fullness (30).

### *Ad libitum* buffet meal

Following the final blood sample collection and VAS at 8 h, participants were provided *ad libitum* access to a cold buffet meal for 30 min in an isolated room with instructions from the researcher to consume food ‘until they felt comfortably full’. Participants were presented with a standardised selection of foods during each visit which included bread, ham, cheese, butter, crisps, apples, bananas, and chocolate. The plate, bowl, and cutlery used and the way in which they were presented were also standardised. All food items were weighed before and after the meal to determine energy and macronutrient intake based on nutritional values provided by the food manufacturers.

### Blood collection and biochemical analysis

Blood samples were obtained in the fasted state and then hourly during each condition via cannulation. Blood was collected into two 4.9 mL EDTA-containing vacuettes (Vacuette, Greiner Bio-One, Austria) to determine circulating concentrations for acylated ghrelin and total PYY. One vacuette was centrifuged at 1500 ***g*** for 10 min at 4°C (Heraeus Multifuge X3R; Thermo Scientific). The plasma supernatant was then aliquoted into separate 1.8 mL cryovials (Fisher Scientific) and stored at −80°C for later analysis of total PYY concentrations. The second vacuette had 40 μL of a potassium phosphate buffer, phydroxymercuribenzoic acid, and sodium hydroxide solution added prior to centrifugation to prevent acylated ghrelin degradation (31). The supernatant was then separated from the precipitate, before 100 μL of 1 M hydrochloric acid was added per mL plasma. The sample was then centrifuged for a further 5 min (31) before being aliquoted into cryovials and stored at −80°C. Hormone concentrations were analysed using commercially available ELISA kits. Samples from each participant were run on the same plate to eliminate inter-assay variation. Precision of each assay was verified by assessing high- and low-concentration quality controls. Intra-assay coefficients of variation for total PYY and acylated ghrelin were 8.9 and 8.3%, respectively.

### Data analysis

Postprandial responses for appetite hormone concentrations were calculated as total area under curve (TAUC) and net incremental area under the curve (iAUC) using the trapezoid method. The curve was characterised by appetite measures (y-axis) plotted over the time points (x-axis). The area under the baseline concentration was subtracted from TAUC to determine iAUC. TAUC was calculated for each subjective appetite outcome and overall appetite. Relative energy intake (REI) was calculated as energy intake for the whole condition minus physical activity energy expenditure (32). Statistical analysis was conducted per protocol. Linear mixed models were performed in SPSS version 22.0 (IBM) to assess differences in the outcomes between experimental conditions. For area under the curve, energy intake, and macronutrient intake outcomes, condition was entered as a fixed factor. A condition × time analysis, with condition and time (categorical) entered as fixed factors, was also conducted due to the differences in the timing and pattern of the physical activity performed in EX-SIT and SIT-ACT. Sidak correction was used for multiple comparisons. Age, sex, body fat %, and baseline values for each outcome measure were included as covariates and entered as fixed factors in all models. Energy intake and relative energy intake models included age, sex, and body fat % as covariates. Participants were entered as a random factor in the models. The data are presented as mean (95% CI). The two-tailed alpha level for significance testing was set as *P* ≤ 0.05. The magnitude of difference between conditions was determined using Cohen’s d effect sizes: small = 0.2, medium = 0.5, and large = 0.8 (33).

## Results

### Participants

Sixteen participants were enrolled in the study. Two participants did not complete any of the three experimental conditions due to not being able to make themselves available for the required times. Fourteen participants (seven female) completed all three experimental conditions. The descriptive characteristics of the participants are shown in Table 1.

**Table 1:** Participant characteristics.

Characteristics	Mean ± s.d.
Age (years)	29.0 ± 9.7
Height (cm)	172.8 ± 5.9
Body mass (kg)	78.5 ± 20.4
Body mass index (kg/m^2^)	26.1 ± 5.8
Body fat (%)	26.1 ± 7.5
Maximum oxygen uptake (mL/kg/min)	38.6 ± 4.2

### Physical activity bout characteristics

As estimated by the Actiheart, overall total physical activity energy expenditure did not differ between the EX-SIT and SIT-ACT conditions (see Table 2). The mean intensity and energy expenditure of the high-intensity physical activity bouts in SIT-ACT were significantly higher than the continuous moderate-intensity exercise bout in EX-SIT.

**Table 2:** Physical activity bout characteristics for the experimental conditions.

	EX-SIT	SIT-ACT	Main effect of condition (*P*)
Total physical activity energy expenditure (kJ)	661 (476, 828)	732 (539, 891)	0.236
Mean physical activity bout intensity (METs)	5.7 (4.7, 6.7)	9.0 (8.0, 10.0)	<0.001
Mean physical activity bout energy expenditure (kJ/min)	22.7 (16.2, 29.2)	36.8 (30.3, 43.4)	<0.001

Data are marginal means (95% CI) for the main effect of condition.

EX-SIT, continuous moderate-intensity physical activity followed by prolonged sitting; METs, metabolic equivalent of task; SIT-ACT, sitting interrupted with high-intensity physical activity breaks.

### Appetite perceptions

There were no significant differences in fasting values for each subjective appetite variable between experimental conditions (see Table 3). For the AUC analyses (see Table 3), the main effect of condition for satisfaction was significant. Total AUC for satisfaction was higher by 16% during SIT-ACT than EX-SIT (*P* = 0.021; d = 0.39, small effect) but was not different to SIT (*P* = 0.888; d = 0.09, trivial effect). There was also no difference between EX-SIT and SIT (*P* = 0.092; d = 0.30, small effect). The main effect of condition for overall appetite TAUC was also significant, with scores being 11% lower in SIT-ACT than EX-SIT (*P* = 0.018; d = 0.35, small effect). Differences in overall appetite were not different between SIT and SIT-ACT (*P* = 0.971; d = 0.02, trivial effect) or SIT and EX-SIT (*P* = 0.265; d = 0.34, small effect). There was no significant main effect of condition for hunger, fullness, or PFC, with effect sizes ranging from d = 0.02 to 0.37.

**Table 3:** Fasting appetite, appetite area under the curve, and energy intake values for each condition.

	SIT	EX-SIT	SIT-ACT	Main effect of condition (*P*)
**Subjective appetite**				
Fasting hunger (mm)	46.6 (33.1, 60.2)	58.9 (45.7, 72.0)	61.6 (49.2, 74.0)	0.089
Fasting satisfaction (mm)	27.7 (16.7, 38.7)	25.5 (17, 34.0)	27.4 (20.9, 33.8)	0.929
Fasting fullness (mm)	33.9 (19.9, 47.8)	25.5 (15.7, 35.3)	24 (16.6, 31.4)	0.204
Fasting PFC (mm)	60.6 (48.9, 72.3)	69.1 (60.9, 77.3)	68.4 (63.1, 73.6)	0.240
Fasting overall appetite (mm)	61.4 (51.3, 71.6)	69.3 (60.3, 78.2)	69.6 (63.7, 75.6)	0.139
Hunger TAUC (mm/8 h)	350.9 (281.6, 420.2)	390.0 (321.5, 458.5)	348.7 (279.9, 417.4)	0.193
Satisfaction TAUC (mm/8 h)	414.2 (334.6, 493.8)	368.5 (288.9, 448.1)	427.3 (347.7, 506.9)^*^	**0.018**
Fullness TAUC (mm/8 h)	407.6 (329.3, 485.9)	369.1 (291.2, 447.1)	419.2 (341.2, 497.2)	0.114
PFC TAUC (mm/8 h)	440.9 (371.8, 510.0)	461.3 (392.4, 530.2)	412.5 (343.6, 481.3)	0.076
Overall appetite TAUC (mm/8 h)	409.9 (336.2, 483.5)	457.1 (383.4, 530.7)	407.7 (334.0, 481.3)^*^	**0.008**
**Energy and macronutrient intake**				
Buffet energy intake (kJ)	3874 (3212, 4536)	3960 (3298, 4621)	3804 (3142, 4466)	0.833
Relative energy intake (kJ)	3852 (3134, 4569)	3290 (2565, 4015)	3092 (2367, 3818)^**^	**0.021**
Carbohydrate (kJ)	1738 (1539, 1937)	1825 (1626, 2023)	1671 (1472, 1870)	0.246
Fat (kJ)	1659 (1281, 2037)	1650 (1272, 2027)	1630 (1252, 2008)	0.982
Protein (kJ)	477 (392, 562)	486 (401, 571)	503 (418, 588)	0.718

Data are marginal means (95% CI) for the main effect of condition. Bold indicates significant main effect.

*Significant difference between SIT-ACT and EX-SIT (*P* < 0.05); **Significant difference between SIT-ACT and SIT (*P* < 0.05).

EX-SIT, continuous moderate-intensity physical activity followed by prolonged sitting; PFC, prospective food consumption; SIT, prolonged sitting; SIT-ACT, sitting interrupted with high-intensity physical activity breaks; TAUC, total area under the curve.

**Figure 2:**
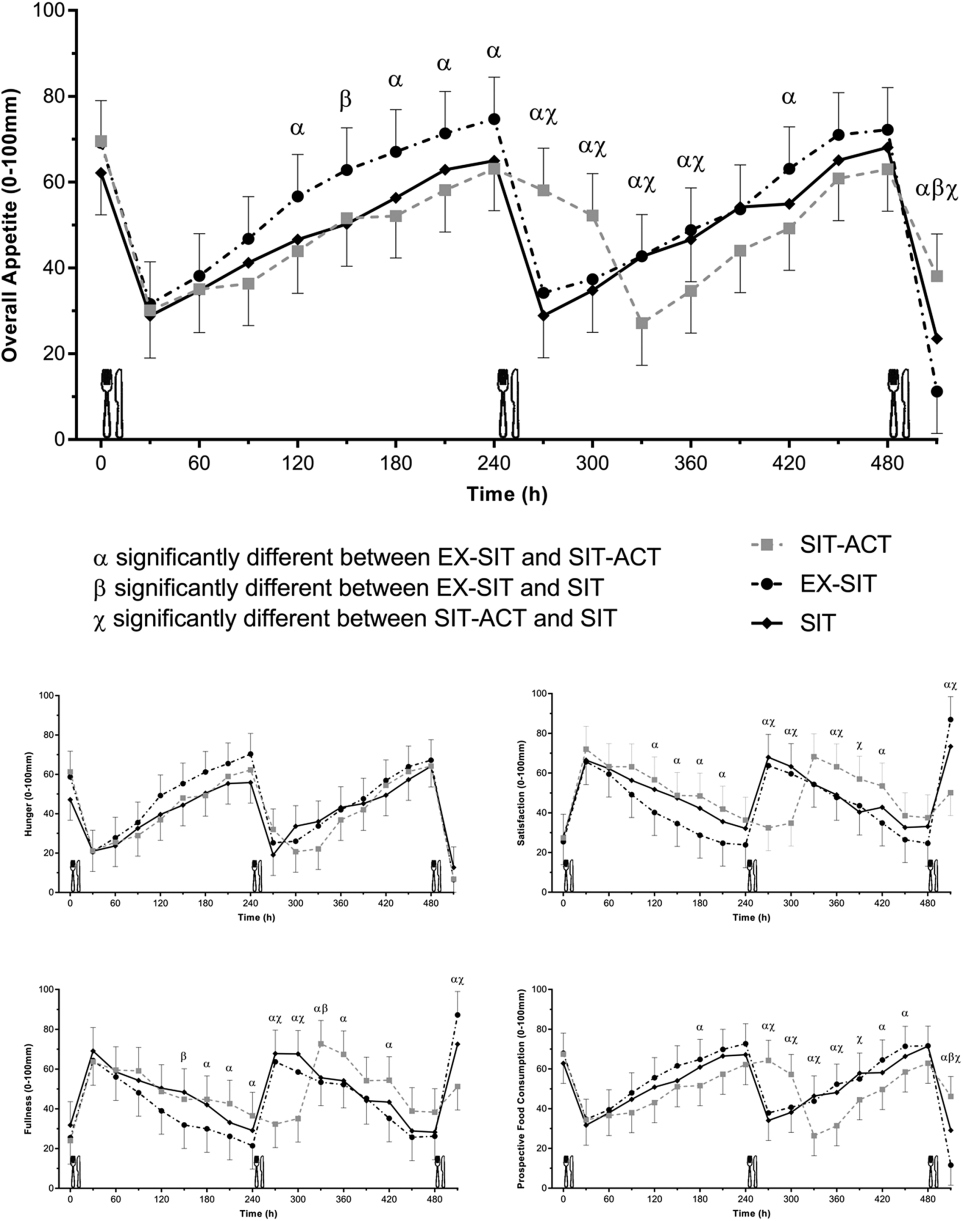
Subjective appetite responses during the experimental conditions.

For the condition × time analyses (see Fig. 2), we report here only the significant effects and between-condition comparisons. The main effect of condition was significant (*P* < 0.001) for hunger with lower values in SIT and SIT-ACT than EX-SIT across the experimental period (*P* = 0.004 and *P* = 0.003, respectively; see Supplementary Table 1, see section on supplementary materials given at the end of this article). The condition × time interaction for hunger was not significant (*P* = 0.175). Satisfaction was lower in EX-SIT than SIT and SIT-ACT (main effect of condition *P* = 0.002 and *P* < 0.001, respectively). The condition × time effect was significant (*P* < 0.001) with higher satisfaction in SIT-ACT than EX-SIT in the post-breakfast time period at 120, 150, 180, and 210 min. In the period shortly after lunch (270–300 min), this was reversed with lower satisfaction in SIT-ACT than EX-SIT and SIT. From 360 to 420 min, satisfaction returned to being higher in SIT-ACT than SIT and EX-SIT. Satisfaction was significantly lower immediately following the buffet meal in SIT-ACT than SIT and EX-SIT. A similar pattern was found for fullness as shown in Fig. 2.

In the condition × time analysis of PFC, there was a main effect of condition with higher values in EX-SIT than in SIT-ACT (*P* = 0.001). The condition × time effect was significant (*P* < 0.001) with significantly lower PFC at 180 min in SIT-ACT than EX-SIT. Values were significantly higher at 270–300 min in SIT-ACT than in SIT and EX-SIT but significantly lower in SIT-ACT than SIT and EX-SIT throughout the rest of the post-lunch period. Immediately post-buffet, PFC was significantly higher in SIT-ACT than SIT and EX-SIT.

Overall appetite during the conditions was significantly higher in EX-SIT than SIT and SIT-ACT (*P* < 0.001), but the significant condition × time interaction (*P* < 0.001) indicated that this effect depended on time. Overall appetite was significantly lower at most time points from 120 to 240 min in SIT-ACT than EX-SIT and significantly higher in EX-SIT than SIT at 150 min. Overall appetite was significantly higher in SIT-ACT than SIT and EX-SIT immediately after lunch (270–300 min) with this being reversed from 330 to 420 min. Immediately after the buffet, overall appetite was significantly higher in SIT-ACT than SIT and EX-SIT and significantly lower in EX-SIT than SIT.

### Appetite hormones

Fasting concentrations for acylated ghrelin and total PYY did not differ significantly between conditions (see Table 4). Postprandial iAUC and TAUC did not differ significantly between conditions for acylated ghrelin or total PYY, as shown in Table 4. Effect sizes were trivial (d = 0.04–0.11) for differences in acylated ghrelin iAUC and TAUC between conditions. For total PYY iAUC, there was a small effect (d = 0.30) for EX-SIT compared with SIT-ACT and for EX-SIT compared with SIT (d = 0.40). For total PYY TAUC, there was a small effect for the differences between EX-SIT and SIT (d = 0.40) and SIT-ACT and SIT (d = 0.35), and a medium effect for the difference between EX-SIT and SIT-ACT (d = 0.74).

**Table 4:** Appetite hormone area under the curve concentrations for each condition.

	SIT	EX-SIT	SIT-ACT	Main effect of condition (*P*)
Fasting acylated ghrelin (pg/mL)	78.4 (49.9, 106.8)	82.5 (54.1, 110.9)	81.2 (52.7, 109.8)	0.738
Fasting total peptide YY (pg/mL)	108.7 (77.8, 139.7)	106.0 (75.1, 136.9)	96.1 (64.2, 128.1)	0.700
Acylated ghrelin iAUC (pg/mL∙8 h)	−55.1 (−142.4, 32.1)	−48.4 (−135.6, 38.9)	−66.6 (−154.4, 21.1)	0.769
Acylated ghrelin TAUC (pg/mL∙8 h)	490.0 (402.7, 577.3)	496.8 (409.5, 584.0)	478.5 (390.7, 566.3)	0.769
Total PYY iAUC (pg/mL∙8 h)	118.8 (−64.9, 302.6)	257.5 (73.8, 441.2)	166.1 (−20.6, 352.8)	0.136
Total PYY TAUC (pg/mL∙8 h)	1004.0 (816.4, 1191.7)	1145.6 (958.4, 1332.8)	873.5 (673.9, 1073.0)	0.125

Data are mean (95% CI).

EX-SIT, continuous moderate-intensity physical activity followed by prolonged sitting; iAUC, incremental area under the curve; SIT, prolonged sitting; SIT-ACT, sitting interrupted with high-intensity physical activity breaks; TAUC, total area under the curve.

**Figure 3:**
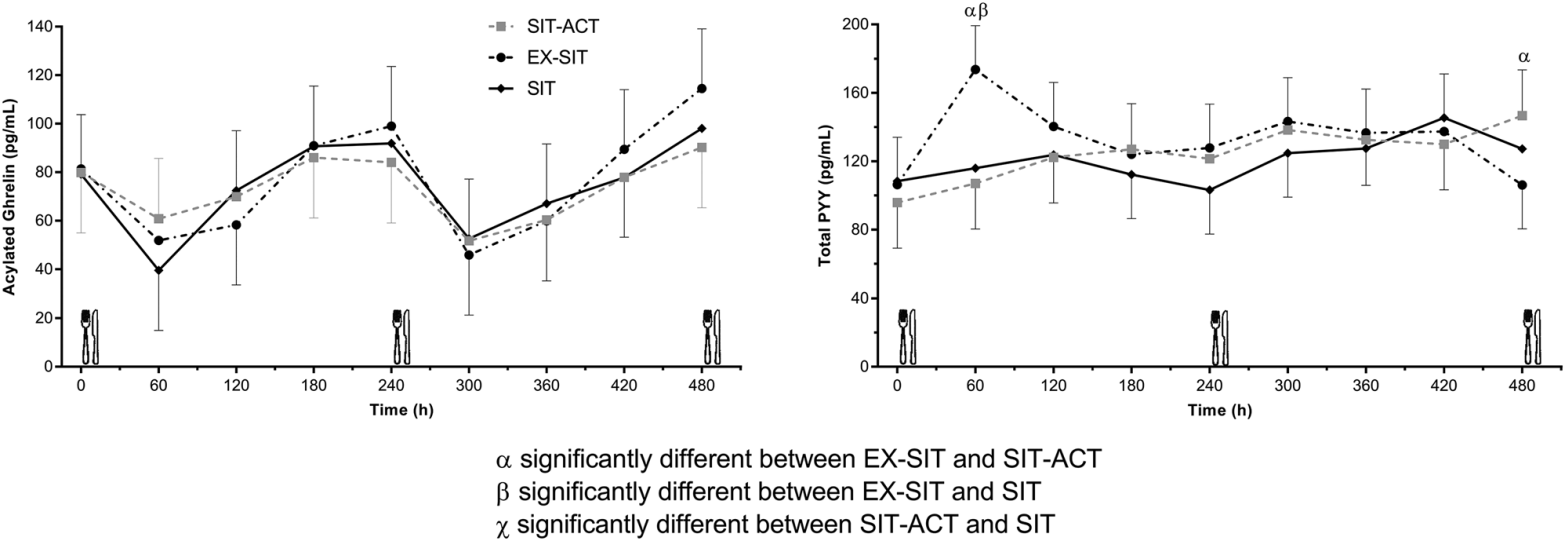
Appetite hormone responses during the experimental conditions.

The main effect of condition (*P* = 0.625) and condition × time interaction (*P* = 0.504) effect was non-significant for mean acylated ghrelin concentrations. For total PYY concentrations, the main effect of condition was not significant (*P* = 0.059; see Supplementary Table 1). The condition × time interaction for total PYY was significant (*P* = 0.014) with higher concentrations in EX-SIT than SIT and SIT-ACT at 60 min (i.e. immediately following the continuous moderate-intensity exercise bout; *P* < 0.001) but higher concentrations in SIT-ACT than EX-SIT at 480 min (*P* = 0.031). Appetite hormone concentrations over time are shown in Fig. 3.

### Energy, macronutrient, and relative energy intake

Energy intake during the *ad libitum* buffet meal did not differ between conditions, with a small effect size (*P* = 0.833, d = 0.06–0.12). Yet, there was a significant main effect of condition for REI, which was lower in SIT-ACT compared with SIT (*P* = 0.024; d = 0.55, medium effect). There were no differences in REI between SIT and EX-SIT (*P* = 0.123; d = 0.41, medium effect) or EX-SIT and SIT-ACT (*P* = 0.851; d = 0.14, small effect). Carbohydrate, fat, and protein intake was not different between conditions (all *P* > 0.367).

## Discussion

The main finding in this study was that breaking up sitting with hourly high-intensity physical activity breaks reduced subjective overall appetite AUC compared to an energy-matched single continuous bout of moderate-intensity exercise but not compared to prolonged sitting in sedentary, healthy adults. This is in line with previous research in which appetite was reduced when adults with obesity engaged in hourly 5-min moderate-intensity walking bouts over 12 h compared with an energy-matched continuous moderate-intensity exercise bout performed in the morning (10). The current study furthers knowledge by extending this finding to high-intensity physical activity breaks. Effects sizes, though, for the lower subjective appetite were small and the meaningfulness of these magnitudes of change requires clarification. Similar to the present study, the moderate-intensity physical activity breaks in the study by Holmstrup, Fairchild, Keslacy, Weinstock, Kanaley (10) did not affect subjective appetite AUC over a single day in comparison to prolonged sitting without physical activity. Other research has reported no changes in appetite in non-overweight adults who engaged in 2-min light or moderate-intensity walking bouts every 20 min over 5 h (13) or 2-min moderate-intensity walking breaks every 30 min over a 2-day period (34). It thus appears that regular physical activity breaks suppress appetite over a single day compared with continuous exercise in participants with obesity and normal weight status but not when compared to prolonged sitting.

Perhaps due to the different pattern of physical activity performed in short, frequent bouts vs in one continuous single session, the direction of some of the between-condition differences in appetite changed over time. Specifically, the high-intensity physical activity breaks performed across the day increased satisfaction and fullness when compared with a single moderate-intensity bout in the period between breakfast and lunch but lowered satisfaction and fullness and increased PFC during the majority of the post-lunch period when compared with continuous moderate-intensity exercise and prolonged sitting. Further, subjective overall (composite) appetite was lower in response to high-intensity physical activity breaks than moderate-intensity exercise in the late morning period specifically, as well as the majority of the post-lunch period. In line with these findings, Holmstrup, Fairchild, Keslacy, Weinstock, Kanaley (10) found that the hunger-suppressing effects of moderate-intensity physical activity breaks compared to prolonged sitting occurred later in the day. This suggests that there may need to be an accumulation of physical activity bouts throughout the day for appetite-suppressing effects to occur post-lunch. These data also highlight the need to assess individual components of appetite, which may respond differently to manipulations in physical activity. In the present study, the continuous moderate-intensity exercise bout suppressed all subjective appetite measures, other than hunger, for short periods after lunch relative to sitting interrupted with high-intensity physical activity breaks but not compared to prolonged sitting. The differences between conditions suggested that continuous exercise led to reduced subjective appetite outcomes immediately following the buffet meal. It could be speculated that the perception of being ‘comfortably full’ during the *ad libitum* buffet meal was different in the continuous exercise condition than in the high-intensity physical activity breaks condition, leading to reduced appetite sensations immediately following the meal despite similar energy and macronutrient intake. Overall, these findings suggest that high-intensity physical activity breaks may provide a sufficient stimulus for appetite suppression across the day.

Although perceptions of overall appetite were suppressed in response to hourly high-intensity physical activity breaks, there was no subsequent effect on *ad libitum* energy intake. This is synonymous with previous research evaluating 2-min light and moderate-intensity walking breaks every 20 min (13) and 2-min moderate-intensity walking breaks every 30 min (34). The present study extends these findings by demonstrating that high-intensity physical activity breaks also do not result in an acute compensatory increase in subsequent energy intake. It is not known whether the hourly moderate-intensity physical activity bouts that suppressed appetite in the study by Holmstrup, Fairchild, Keslacy, Weinstock, Kanaley (10) affected *ad libitum* energy intake, as this was not evaluated. As physical activity energy expenditure between the activity conditions was matched in the current study, the similar *ad libitum* energy intake meant that REI was significantly lower by 921 kJ (~220 kcal) in the hourly high-intensity physical activity breaks vs prolonged sitting condition; an effect not seen in response to the continuous moderate-intensity exercise bout. The medium effect size for this difference suggests that the lower REI is potentially meaningful. If repeated over the long term, this ‘energy gap’ could aid with obesity prevention and, to a certain degree, weight loss maintenance (35). Indeed, this is more than double the 100 kcal/day energy deficit that has been recommended to prevent excess weight gain in 90% of the US population (35). That said, as energy intake compensation is often slow (detectable after around 2 weeks) and variable between individuals (36), longer-term trials are required to determine the implications of this finding for weight management.

Appetite hormone responses were not congruent with the suppressed subjective appetite in response to high-intensity physical activity breaks relative to continuous moderate-intensity exercise across the day and prolonged sitting during the majority of the post-lunch period. Although total PYY concentrations did not differ significantly between conditions, the small effect size for higher concentrations following continuous moderate-intensity exercise suggests potentially meaningful differences. The finding that high-intensity physical activity breaks increased satiety, but had little effect on PYY, is similar to a prior study investigating responses to moderate-intensity physical activity breaks (10). The higher intensity of the physical activity in the present study may be expected to result in greater changes in gut hormones to promote satiety (15), but this occurred only at the end of the condition, indicating that a certain overall duration of physical activity may be required to elicit suppressions in total PYY. There is also doubt with regard to the extent by which gut hormones regulate appetite, which may be due to intra-individual variability in appetite-related variables (37). Previous research has reported significant reductions in PYY and increases in acylated ghrelin in response to moderate and high-intensity exercise that is completed in single bouts lasting 30–90 min, which may act as physiological mediators in appetite suppression (9). The small and medium effect sizes explaining higher PYY iAUC and TAUC in response to continuous moderate-intensity exercise compared with high-intensity physical activity breaks and prolonged sitting support these findings. However, the evidence is not consistent (38, 39). With regard to physical activity breaks, their short duration and intermittent nature may be insufficient to cause alterations in circulating appetite-regulating hormones in this sample of healthy individuals. In fact, the present data suggest there may be potentially meaningful, albeit non-significant, reductions in PYY concentrations in response to high-intensity physical activity breaks compared with prolonged sitting. Thus, alternative physiological and psychological mechanisms may explain the suppression of appetite in response to physical activity breaks for example, delayed gastric emptying and a reduction in motivation to eat (36).

In terms of real-life application, the short duration (2 min 32 s) and relatively infrequent (hourly) nature of the physical activity breaks in this study could be considered a more feasible and practical strategy compared with longer duration structured exercise or more regular physical activity breaks (e.g. every 20–30 min) (13, 34), particularly for those who lack motivation to engage in structured exercise sessions. In this study, participants were at a brisk walk to slow jogging pace in order to elicit a high-intensity treadmill speed. At home or in the workplace, a similar exertion could be achieved by, for example, stair climbing or pacing up and down a corridor. The feasibility and effectiveness of high-intensity physical activity breaks in real-world settings should be investigated to inform weight management strategies.

The strengths of this study include the randomised crossover design, control and standardisation procedures before and during the experimental conditions, and the range of subjective and objective appetite-related outcomes. A limitation of the study is that it was not possible to isolate the effects of physical activity frequency/pattern on the outcomes due to the physical activity conditions not being matched for intensity (as confirmed via continuous physical activity energy expenditure assessment) or duration. It may have not been feasible for sedentary participants to engage in a continuous high-intensity exercise bout that matched the total volume of physical activity in the physical activity breaks condition; thus, the physical activity characteristics of the interventions may be favourable in terms of ecological validity. Also, total PYY was measured as opposed to PYY_3-36_, which is a more potent appetite suppressant (40). That said, changes in total PYY are likely to reflect changes in PYY_3-36_ as these two variables are strongly correlated (41). There were also no exclusion criteria with respect to current dieting or recent extreme changes in body mass, which could influence appetite regulation. During the experimental conditions, it is possible that participants were exposed to food cues during the activities they were permitted to undertake (e.g. using a phone), which could have affected their subjective appetite. However, it could be expected that participants would undertake similar activities across the conditions as the same instructions were provided, therefore minimising this bias. Lastly, the participants were sedentary but otherwise healthy, which may limit generalisability to clinical populations of individuals who are overweight or obese.

In conclusion, breaking up sitting with hourly high-intensity physical activity breaks acutely reduced subjective appetite over a single day when compared to a continuous energy-matched moderate-intensity exercise bout. The findings also suggest that high-intensity physical activity breaks suppress subjective appetite during the majority of the post-lunch lunch period compared to prolonged sitting. Furthermore, the increased energy expenditure from the high-intensity physical activity breaks created an energy deficit that was not compensated for during subsequent food intake. Thus, breaking up sitting with high-intensity physical activity may represent an effective alternative to traditional structured continuous exercise to help with obesity prevention and weight management strategies.

## Supplementary Material

Supplementary Table 1 Subjective appetite and appetite hormone concentrations for each condition

## Declaration of interest

The authors declare there is no conflict of interest that could be perceived as prejudicing the impartiality of the research reported.

## Funding

This work was supported by funding from the Society for Endocrinology.
