# Supplementary material for: Short, frequent high-intensity physical activity breaks reduce appetite compared to a continuous moderate-intensity exercise bout
Source: Endocr Connect. 2022 Dec 22;12(2):e220259. doi: 10.1530/EC-22-0259 (PMC9874970; doi:10.1530/EC-22-0259)
Supplement: Supplementary Table 1 Subjective appetite and appetite hormone concentrations for each condition [file supplementary_table_1.pdf]

Supplementary Table 1 Subjective appetite and appetite hormone concentrations for each condition

	SIT	EX-SIT	SIT-ACT	Main effect of condition (P)
Hunger (mm)	40.6 (33.0, 48.1)*	45.2 (37.6, 52.7)	40.7 (33.1, 48.2)*	**<0.001**
Satisfaction (mm)	48.8 (39.9, 57.7)*	44.1 (35.2, 53.0)	49.6 (40.7, 58.5)*	**<0.001**
Fullness (mm)	48.8 (39.5, 58.2)*	43.9 (34.5, 53.2)	48.2 (38.9, 57.6)*	**<0.001**
PFC (mm)	51.5 (43.8, 59.1)	53.5 (45.9, 61.1)	49.0 (41.4, 56.6)*	**0.002**
Overall appetite (mm)	48.1 (40.3, 56.0)*	52.9 (45.1, 60.7)	48.2 (40.4, 56.0)*	**<0.001**
Acylated ghrelin (pg/mL)	74.4 (51.3, 97.4)	76.8 (53.7, 99.9)	73.4 (50.4, 96.5)	0.625
Total peptide YY (pg/mL)	120.9 (102.9, 138.9)	132.8 (114.8, 150.8)	124.6 (106.4, 142.7)	0.058

Data are marginal means (95% CI) for the main effect of condition. SIT, prolonged sitting; EX-SIT, continuous moderate-intensity physical activity followed by prolonged sitting; SIT-ACT, sitting interrupted with high-intensity physical activity breaks; PFC, prospective food consumption. Bold indicates significant main effect. *Significantly different compared with EX-SIT ($p \leq 0.05$).
